# Supplementary material for: Structural and electronic properties of epitaxial multilayer h-BN on Ni(111) for spintronics applications
Source: Sci Rep. 2016 Mar 24;6:23547. doi: 10.1038/srep23547 (PMC4806377; doi:10.1038/srep23547)
Supplement: Supplementary Information [file srep23547-s1.pdf]

Supplementary material for manuscript:

Structural and electronic properties of epitaxial multilayer *h*-BN on Ni(111) for spintronics applications

A. A. Tonkikh,[1,2] E. N. Voloshina,[3] P. Werner,[1] H. Blumtritt,[1] B. Senkovskiy,[4,5] G. Güntherodt,[1,6] S. S. P. Parkin,[1] and Yu. S. Dedkov[7]

[1]Max Planck Institute of Microstructure Physics, Weinberg 2, 06120 Halle (Saale), Germany

[2]Institute for Physics of Microstructures RAS , 603950, GSP-105, Nizhny Novgorod, Russia

[3]Humboldt-Universität zu Berlin, Institut für Chemie, 10099 Berlin, Germany

[4]Institute of Solid State Physics, Dresden University of Technology, 01062 Dresden, Germany

[5]St. Petersburg State University, 198504 St. Petersburg, Russia

[6]2nd Institute of Physics and JARA-FIT, RWTH Aachen University, 52074 Aachen, Germany

[7]SPECS Surface Nano Analysis GmbH, Voltastraße 5, 13355 Berlin, Germany

List of tables and figures:

Fig. S1. RHEED images of (a) Ni(111) substrate, (b) Ni buff-layer on Ni(111), and (c) a 10 ML-thick *h*-BN film on Ni(111).

Fig. S2. Angular resolved XPS spectra (Ni $2p_{3/2}$ and N $1s$) at different take-off angles θ.

Table I Mean interlayer distances in the ml-h-BN/Ni(111) system for different thickness of the BN layer

Fig. S3. Structure of the ml-h-BN/Ni(111) system where h-BN has an A-B stacking. Distances used in Table I are marked in the figure.

Fig. S4. Layer resolved partial density of states (PDOS) for 1 ML-h-BN/Ni(111) (spin-up – top panels; spin-down – bottom panels).

Fig. S5. Layer resolved partial density of states (PDOS) for 2 ML-h-BN/Ni(111) (spin-up – top panels; spin-down – bottom panels).

Fig. S6. Layer resolved partial density of states (PDOS) for 3 ML-h-BN/Ni(111) (spin-up – top panels; spin-down – bottom panels).

Fig. S7. Layer resolved partial density of states (PDOS) for 4 ML-h-BN/Ni(111) (spin-up – top panels; spin-down – bottom panels).

Fig. S8. Layer resolved partial density of states (PDOS) for 5 ML-h-BN/Ni(111) (spin-up – top panels; spin-down – bottom panels).

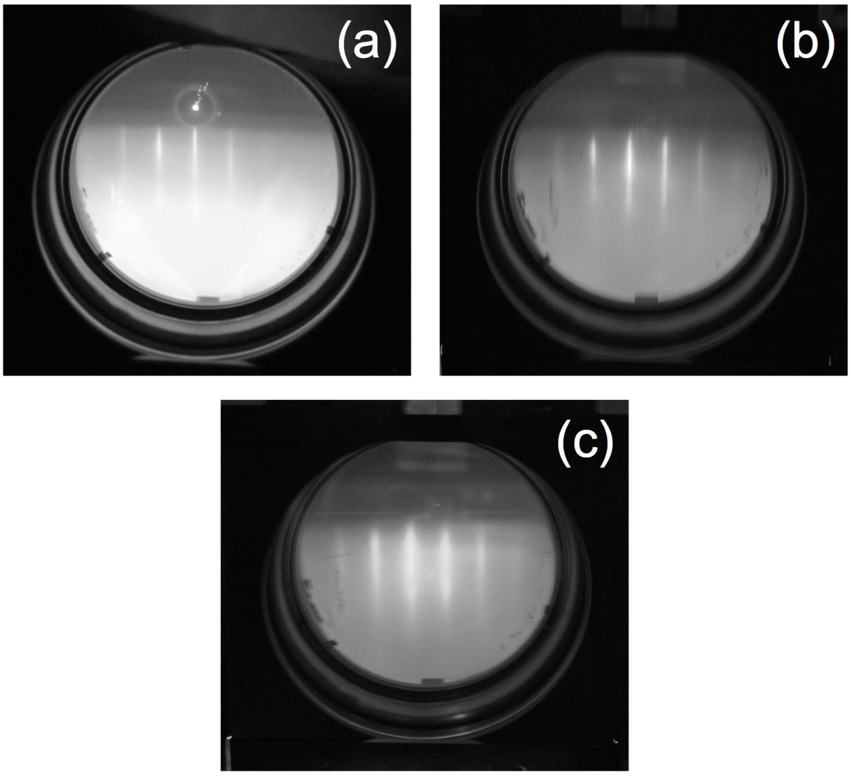

Fig. S1. RHEED images of (a) Ni(111) substrate, (b) Ni buff-layer on Ni(111), and (c) a 10 ML-thick *h*-BN film on Ni(111).

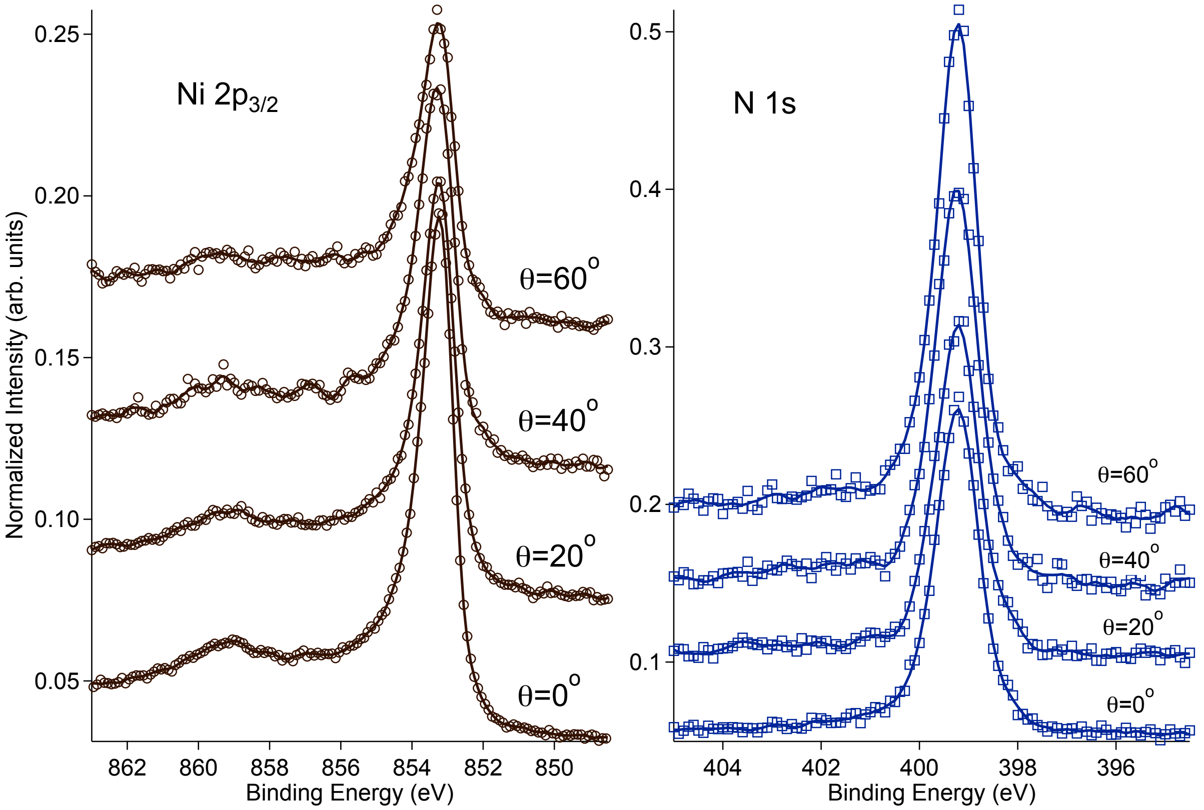

Fig. S2. Angular resolved XPS spectra (Ni $2p_{3/2}$ and N $1s$) at different take-off angles θ.

TABLE I: Mean interlayer distances in the ml-h-BN/Ni(111) system for different thickness of the BN layer (see Fig. S1).

	1ML BN/Ni	2ML BN/Ni	3ML BN/Ni	4ML BN/Ni	5ML BN/Ni
d_1	2.04	2.04	2.04	2.04	2.04
d_2	2.02	2.02	2.02	2.02	2.02
d_3	2.00	2.00	2.00	2.00	2.00
d_4	2.04	2.03	2.03	2.03	2.03
d_5		3.01	3.00	3.00	3.00
d_6			3.06	3.05	3.05
d_7				3.06	3.05
d_8					3.06

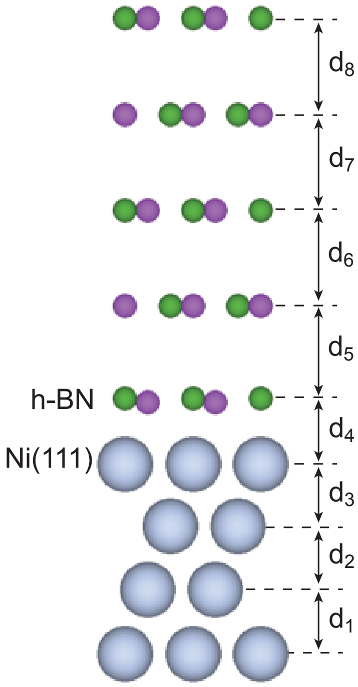

Fig. S3. Structure of the ml-h-BN/Ni(111) system where h-BN has an A-B stacking. Distances used in Table I are marked in the figure.

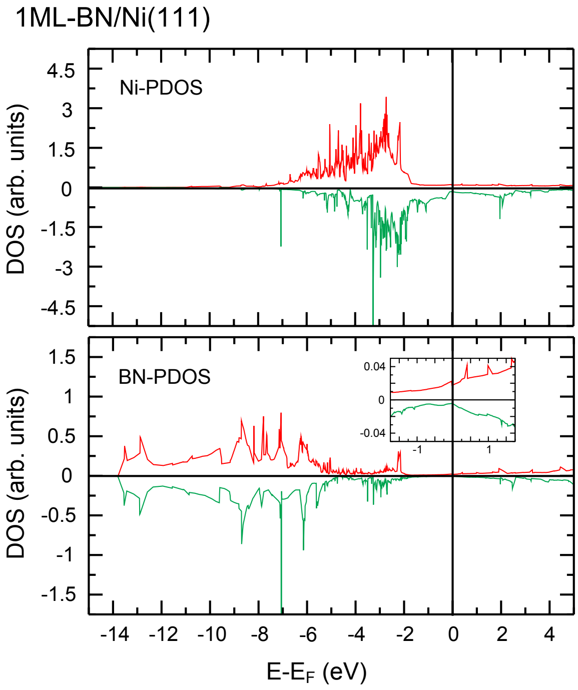

Fig. S4. Layer resolved partial density of states (PDOS) for 1 ML-h-BN/Ni(111) (spin-up – top panels; spin-down – bottom panels).

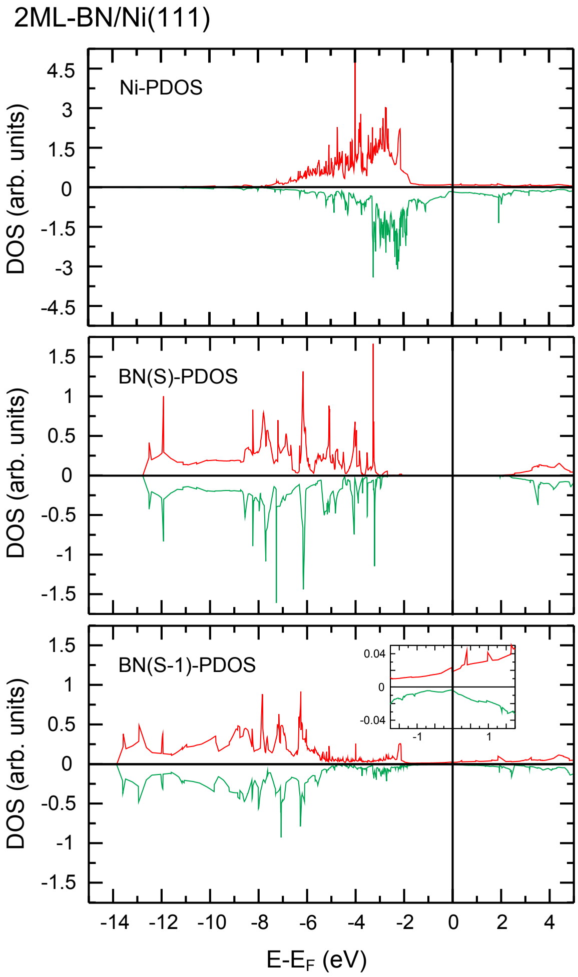

Fig. S5. Layer resolved partial density of states (PDOS) for 2 ML-h-BN/Ni(111) (spin-up – top panels; spin-down – bottom panels).

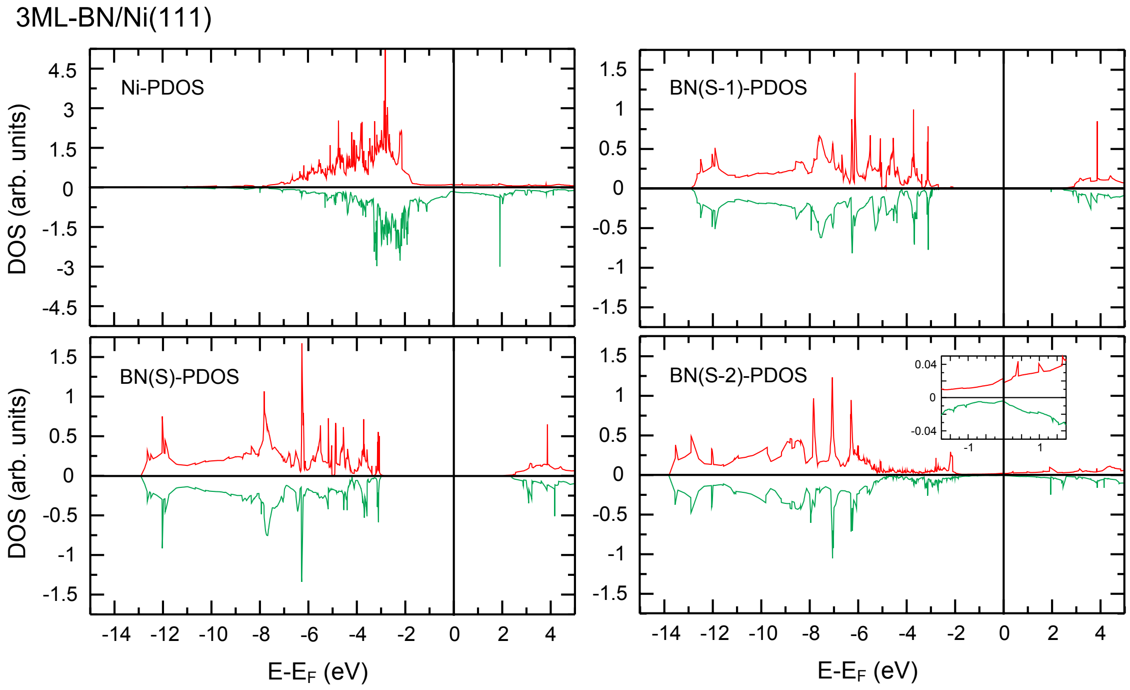

Fig. S6. Layer resolved partial density of states (PDOS) for 3 ML-h-BN/Ni(111) (spin-up – top panels; spin-down – bottom panels).

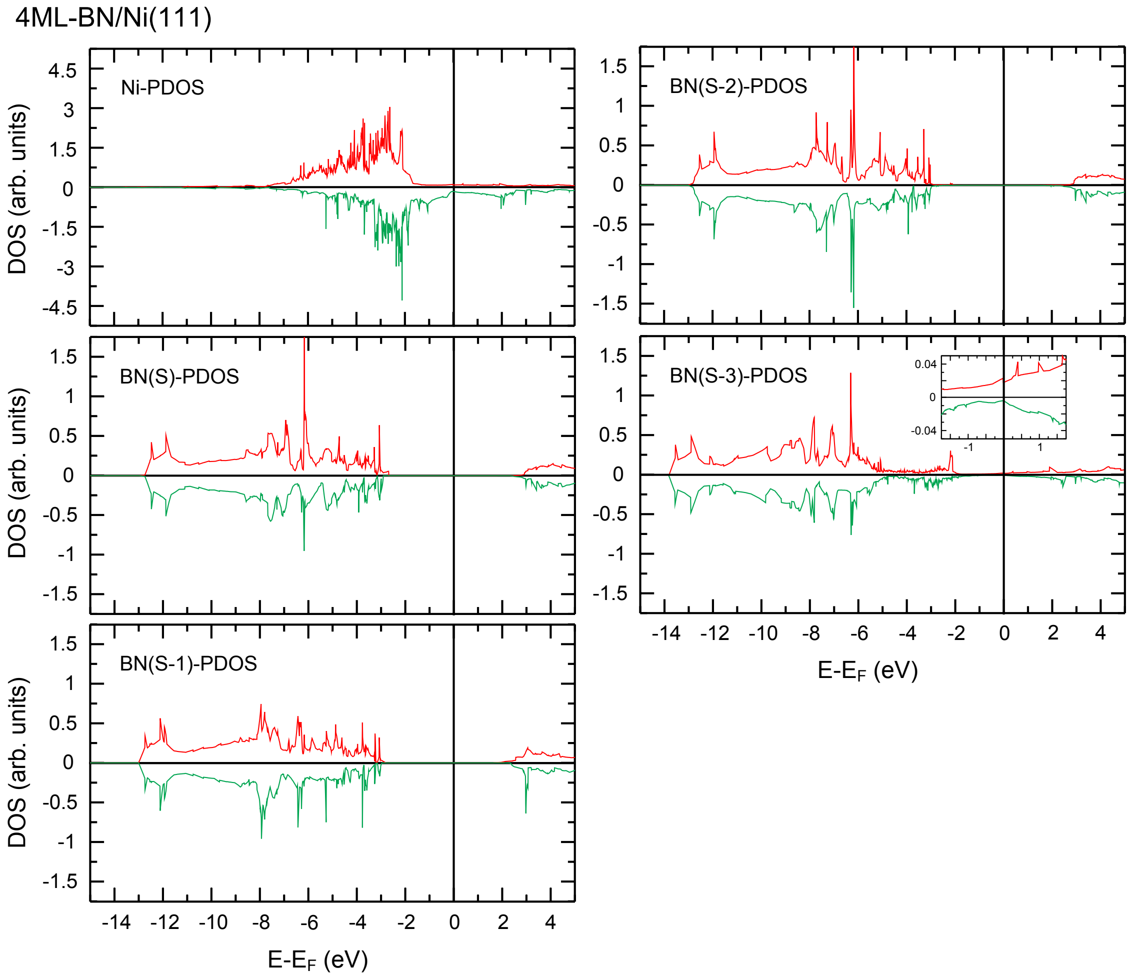

Fig. S7. Layer resolved partial density of states (PDOS) for 4 ML-h-BN/Ni(111) (spin-up – top panels; spin-down – bottom panels).

5ML-BN/Ni(111)

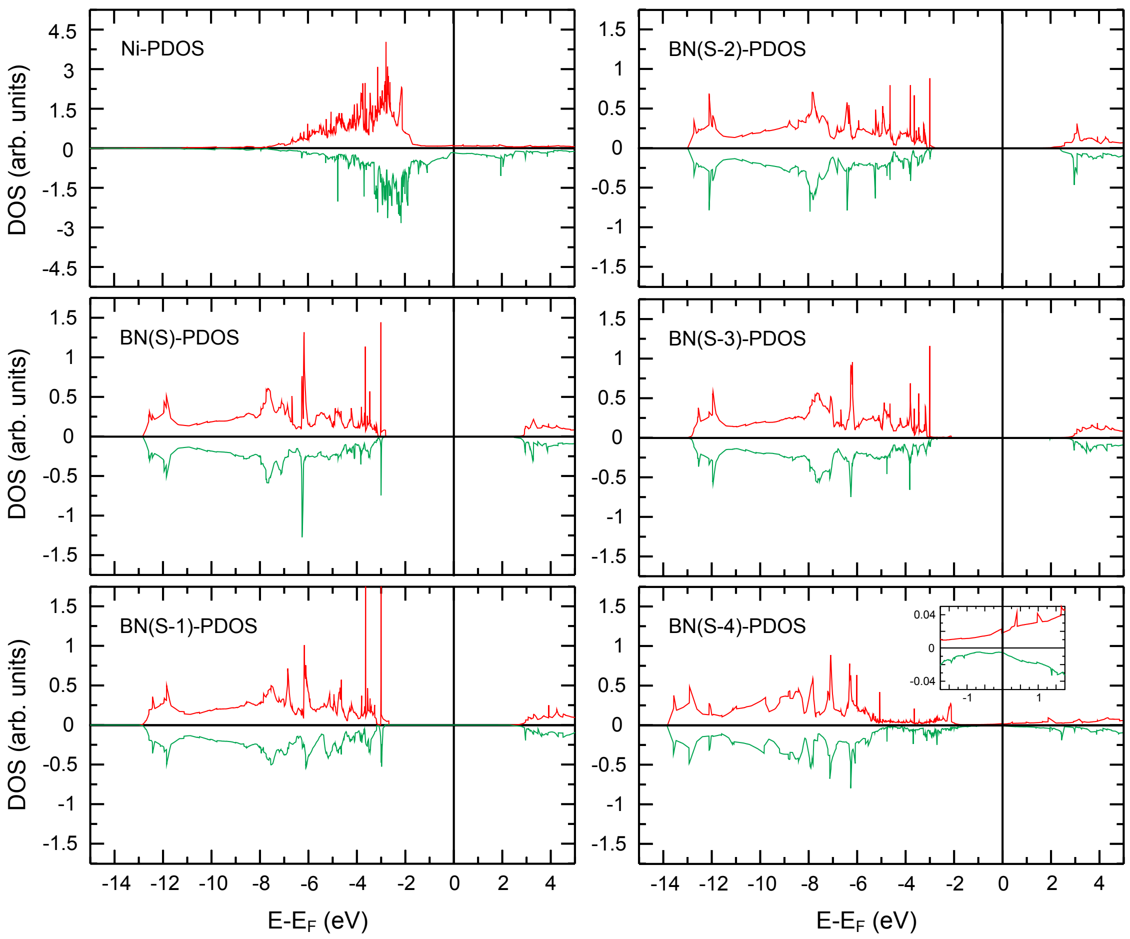

Fig. S8. Layer resolved partial density of states (PDOS) for 5 ML-h-BN/Ni(111) (spin-up – top panels; spin-down – bottom panels).
